# Using gene expression profiles from peripheral blood to identify asymptomatic responses to acute respiratory viral infections

**DOI:** 10.1186/1756-0500-3-264

**Published:** 2010-10-20

**Authors:** Alexander Statnikov, Nikita I Lytkin, Lauren McVoy, Jörn-Hendrik Weitkamp, Constantin F Aliferis

**Affiliations:** 1 Center for Health Informatics and Bioinformatics, New York University School of Medicine, New York, NY 10016, USA; 2 Department of Medicine, New York University School of Medicine, New York, NY 10016, USA; 3 Department of Pathology, New York University School of Medicine, New York, NY 10016, USA; 4 Division of Neonatology, Department of Pediatrics, Vanderbilt University School of Medicine and Monroe Carell Jr. Children's Hospital at Vanderbilt, Nashville, TN 37232, USA; 5 Department of Biostatistics, Vanderbilt University, Nashville, TN, 37232, USA

## Abstract

**Background:**

A recent study reported that gene expression profiles from peripheral blood samples of healthy subjects prior to viral inoculation were indistinguishable from profiles of subjects who received viral challenge but remained asymptomatic and uninfected. If true, this implies that the host immune response does not have a molecular signature. Given the high sensitivity of microarray technology, we were intrigued by this result and hypothesize that it was an artifact of data analysis.

**Findings:**

Using acute respiratory viral challenge microarray data, we developed a molecular signature that for the first time allowed for an accurate differentiation between uninfected subjects prior to viral inoculation and subjects who remained asymptomatic after the viral challenge.

**Conclusions:**

Our findings suggest that molecular signatures can be used to characterize immune responses to viruses and may improve our understanding of susceptibility to viral infection with possible implications for vaccine development.

## Background

Acute respiratory viral infections cause significant morbidity and mortality in the United States and worldwide. Currently clinicians do not have practical means to make a timely and accurate diagnosis of acute viral respiratory infections and often resort to unnecessary antibiotic treatment, which increases healthcare costs and facilitates development of antibiotic resistance. Recently a novel approach was proposed for the diagnosis of acute respiratory infections based on microarray gene expression profiles from peripheral blood samples from human subjects [1]. Using human viral challenge studies with live human rhinovirus (HRV), respiratory syncytial virus (RSV), and influenza A, Zaas *et al. *developed an "acute respiratory viral response" 30-gene panviral signature that accurately identified symptomatic patients with viral infection. The same study also reported that gene expression profiles of subjects prior to viral inoculation (at baseline) were indistinguishable from profiles of subjects who received viral challenge but remained asymptomatic and uninfected. Given the high sensitivity of microarray technology, we were intrigued by the latter result and hypothesize that it was an artifact of data analysis. Since the gene expression dataset of Zaas *et al. *was deposited in the Gene Expression Omnibus, we were able to verify our hypothesis and discover significant differences between these two groups of samples. We demonstrate this by developing a molecular signature that discriminates with high accuracy between uninfected subjects at baseline (prior to viral inoculation) and asymptomatic subjects at the time correlating to peak symptoms in the symptomatic group. This finding has important implications for a better understanding of the complex human immune response to viral antigens. Genes that are differentially expressed in the two groups may provide important clues about decreased susceptibility to viral challenge, which could result in more effective vaccine development or novel therapeutic strategies.

## Methods

To arrive at our findings, we reanalyzed the gene expression data of Zaas *et al. *that contains measurements of 22,277 oligonucleotide probes for 56 blood samples from uninfected subjects (measured at baseline, prior to inoculation) and 30 blood samples from subjects who received viral challenge but remained asymptomatic (measured at corresponding peak symptoms time specific for each virus). We used an improved data-analytic protocol that avoids selection of redundant and biologically irrelevant genes and at the same time maximizes predictive accuracy of the signature [2]. This protocol has recently allowed us to develop a highly accurate and compact molecular signature for differentiation between uninfected subjects and those with acute respiratory viral infections. This signature had high reproducibility as evidenced by its nearly perfect accuracy in the independent data of Ramilo *et al. *[3], and was comprised of genes that are involved in the host immune response [2].

Our data-analytic protocol first involved selection of genes by GLL-PC, a supervised multivariate biomarker discovery method that provably discovers genes in the local pathway around the response variable of interest [4,5]; additional details about GLL-PC are provided in [Additional file 1]. Next, Support Vector Machine (SVM) classifiers with linear kernel and penalty hyper-parameter *C *= 100 were fitted on the selected genes [6]. In order to obtain an unbiased estimate of predictive accuracy that will hold in applications of this molecular signature on future subjects, gene selection and SVM classifier training were performed by stratified 10-fold cross-validation repeated 100 times for different splits of subject into 10 folds [2,7]. Finally, we ensured signature reproducibility and assessed its statistical significance by using a permutation test with significance level α = 0.05 and 10,000 permutations [8].

## Results and Discussion

The data-analytic protocol described above yields an unbiased estimate of predictive accuracy = 0.85 area under ROC curve (AUC); 95% confidence interval [0.76; 0.94] AUC. On average GLL-PC selected 7 genes depending on the training set of cross-validation. Genes selected by GLL-PC in more than 20% of the training sets are listed in Table 1. Genes that were selected in a smaller fraction of training sets may be artifacts of small sample size. Next, GLL-PC and SVM were applied on the entire set of samples, resulting in a 6-gene signature comprised of genes *EIF2S1*, *ZNF91*, *RBM3*, *ATP5S*, *TPPP3*, *GPR97*. Note that all these six genes were also among the top seven most frequently selected by GLL-PC during cross-validation (Table 1), which demonstrates the stability of this gene set despite the inherent heterogeneity of gene expression across subjects.

**Table 1:** Most frequently selected genes by GLL-PC during cross-validation.

*Probe set ID*	*Gene symbol*	*Gene name*	*Frequency of selection by GLL-PC during cross-validation*
201143_s_at	***EIF2S1***	eukaryotic translation initiation factor 2, subunit 1 alpha, 35kDa	99%

218876_at	***TPPP3***	tubulin polymerization-promoting protein family member 3	95%

206059_at	***ZNF91***	zinc finger protein 91	94%

208319_s_at	***RBM3***	RNA binding motif (RNP1, RRM) protein 3	74%

220404_at	***GPR97***	G protein-coupled receptor 97	67%

202124_s_at	*TRAK2*	trafficking protein, kinesin binding 2	58%

213995_at	***ATP5S***	ATP synthase, H+ transporting, mitochondrial F0 complex, subunit s (factor B)	32%

208650_s_at	*CD24*	CD24 molecule	20%

Genes that were also selected by GLL-PC on the entire set of samples are shown with bold

Of the eight genes shown in Table 1, four are known to be involved in the host immune response. *EIF2S1 *encodes the alpha subunit of the translation initiation factor eIF2 complex (eIF2α), which initiates protein synthesis. This protein appears to be crucial for the survival of virally infected cells [9]. The increase of expression of *EIF2S1 *that is observed in the data could mean that host protein synthesis is turned on as response to viral exposure. On the other hand, many viruses, including influenza A viruses, have developed mechanisms favoring the translation of viral over cellular mRNAs leading to a switch from cellular to viral protein synthesis, while inhibiting the cell-encoded antiviral pathways [10]. *RBM3 *encodes a protein that is a member of the heterogeneous nuclear ribonucleoproteins (hnRNPs) that appears to enhance global protein synthesis [11] but also has a role in viral transcription and replication [12]. The *ZNF91 *gene family is a subset of the Krüppel-associated box (KRAB)-containing group of zinc finger genes. Zinc-finger proteins containing the KRAB domain are transcriptional regulators that have been associated with suppression of viral proliferation [13]. The related transcriptional factor *OTK18 *has been shown to be induced by and to suppress HIV-1 infection in mononuclear cells [14]. Finally, *CD24 *is involved in the CD24-Siglec G pathway that protects the host against a fatal reaction to pathological cell death and discriminates danger- versus pathogen-associated molecular patterns [15].

To the best of our knowledge, the remaining 4 genes from Table 1 (*TPPP3*, *GPR97*, *ATP5S*, *TRAK2*) have not yet been specifically associated with host immune responses, however this does not rule them out as novel pharmaceutical targets. Some of these genes are involved in normal cellular function and for some there is evidence of their association with host immune responses: *TPPP3 *encodes members of the tubulin family of proteins, and one study has shown an increase of tubulin in influenza infection [16]. *GPR97 *encodes a member of G protein-coupled receptors that bind chemokines on the surface of immune cells. Chemokine signaling is critical for effective antiviral immune response activation. *ATP5S *encodes a subunit of mitochondrial ATP synthase, and it has been suggested that an increase in mitochondrial activity plays a relevant role in viral replication [17]. *TRAK2 *encodes a trafficking factor that facilitates expression of potassium channels to the cell surface [18].

In further analysis, we identify a likely reason that precluded Zaas *et al. *from detecting a difference between uninfected subjects at baseline and asymptomatic subjects at corresponding peak time. The study of Zaas *et al. *used 30 genes from the "acute respiratory viral response" signature (that was designed for differentiation of *symptomatic *subjects from *uninfected *individuals) to perform a *different *classification task. It is therefore not surprising that even though these 30 genes were predictive for diagnosis of symptomatic subjects from uninfected individuals, they were not predictive for differentiating uninfected subjects at baseline from asymptomatic subjects at the time corresponding to peak symptoms in the symptomatic group. We have verified this assertion by running the original data analysis software of Zaas *et al. *that resulted in random predictive accuracy (0.50 AUC) when using 30 genes from the "acute respiratory viral response" signature and a non-trivial predictive accuracy (0.66 AUC) when using genes selected *specifically *for the classification task of interest. The remainder of the discrepancy between performance of the protocol of Zaas *et al. *and one used in the present study is due to the choice of methods for gene selection, classification, and accuracy estimation (cross-validation). If we substitute sparse probit regression with SVMs in the analysis protocol of Zaas *et al.*, the predictive accuracy increases to 0.75 AUC. If we also substitute factor analysis-based gene selection with GLL-PC and use repeated 10-fold cross-validation, we obtain the result identical to our analysis, i.e. 0.85 AUC.

## Conclusions

In summary, upon reanalysis, the data provided in the study by Zaas *et al. *[1] demonstrate that there is a difference between gene expression profiles of the uninfected subjects prior to viral exposure and the asymptomatic subjects after the exposure. This observation is important for understanding host immune response and warrants validation in independent gene expression data and/or with RT-qPCR. A more detailed understanding of molecular factors that enable some exposed subjects to avoid infection or remain asymptomatic after the exposure while others demonstrate clinical illness could provide targets for development of more effective vaccines and antiviral treatments. More studies should be undertaken to better characterize the gene expression changes in specific viral infections and patient populations.

## Competing interests

The authors declare that they have no competing interests.

## Authors' contributions

Conceived and designed the experiments: NIL, AS, CFA. Performed the experiments: NIL, AS. Analyzed the results of experiments: NIL, AS, CFA, JHW, LM. Wrote the paper: NIL, AS, CFA, JHW, LM. All authors read and approved the final manuscript.

## Supplementary Material

Additional file 1 **GLL-PC and one of its instantiations - Semi-interleaved HITON-PC**. This file contains a description of the supervised biomarker discovery framework GLL-PC and one of its instantiations, termed Semi-interleaved HITON-PC, that was used in this work. Click here for file
