# Supplementary material for: Using gene expression profiles from peripheral blood to identify asymptomatic responses to acute respiratory viral infections
Source: BMC Res Notes. 2010 Oct 20;3:264. doi: 10.1186/1756-0500-3-264 (PMC2975649; doi:10.1186/1756-0500-3-264)
Supplement: Additional file 1 — GLL-PC and one of its instantiations - Semi-interleaved HITON-PC. This file contains a description of the supervised biomarker discovery framework GLL-PC and one of its instantiations, termed Semi-interleaved HITON-PC, that was used in this work. [file 1756-0500-3-264-S1.DOC]

**Additional file 1**

We performed gene selection with Semi-interleaved HITON-PC, an instance of the generative algorithm GLL-PC [4,5] (Tables S1 and S2). The algorithm receives on input a gene expression dataset and a response variable indicating whether a subject is uninfected at baseline or asymptomatic at corresponding peak time. The algorithm outputs genes in the putative local pathway (i.e., the set of parents and children or direct causes and effects) of the response variable. Under fairly broad assumptions, GLL-PC provably discovers genes that are located in the local pathway of the response variable [4,5]. Genes in the local pathway are important because they are likely to yield highest accuracy predictions of the response variable, while other genes do not contribute additional predictive information beyond what is contained in the local pathway. In the present study, GLL-PC was run without symmetry correction (steps 2 and 3 of GLL-PC), with the Fisher’s *Z*-test for vanishing partial correlations (as a test of conditional independence) at significance level α = 5%, and with parameter *max-k* = 3.

**Table S1:** High-level outline and main components (underlined) of the GLL-PC generative algorithm. ***V*** denotes the set of all variables (genes) in the dataset. *T* is the response variable.

| **GLL-PC:** High-level pseudocode and main components of the Generalized Local Learning-Parents and Children (GLL-PC) generative algorithm. The algorithm returns the set of parents and children of *T*, denoted as *PC*(*T*).   1. ***U*** 🡨 GLL-PC-nonsym(*T*) 2. For all *X* ∈ ***U*** 3. If *T* ∉ GLL-PC-nonsym(*X*) then ***U*** 🡨 ***U*** \ {*X*} 4. Return ***U***   **GLL-PC-nonsym(***T***):** Returns *TPC*(*T*), an approximation (“tight” superset) of the set *PC*(*T*).  1. Initialization  a. Initialize a set of candidates for the true *PC*(*T*) set:  *TPC(T)* 🡨 *S*, such that *S* ⊆ ***V*** *\* {*T*}  b. Initialize a priority queue of variables to be examined for inclusion in *TPC(T)*: *OPEN* 🡨 ***V*** *\* {*T* ∪ *TPC*(*T*)}  2. Apply inclusion heuristic function  a. Prioritize variables in *OPEN* for inclusion in *TPC*(*T*)  b. Throw away non-eligible variables from *OPEN*  c. Insert in *TPC*(*T*) the highest-priority variable(s) in *OPEN* and remove them from *OPEN*  3. Apply elimination strategy to remove variables from *TPC*(*T*)  4. Apply interleaving strategy by repeating steps #2 and #3 until a termination criterion is met  5. Return *TPC*(*T*) |
| --- |

**Table S2:** Semi-interleaved HITON-PC, as an instance of the generative algorithm GLL-PC.

| **Semi-Interleaved HITON-PC:** This algorithm is derived from GLL-PC with the following instantiation specifics:  Initialization  *TPC*(*T*) 🡨 ∅  Inclusion heuristic function   1. Sort in descending order the variables *X* in *OPEN* according to their pairwise association with *T* 2. Remove from *OPEN* variables with zero association with *T* 3. Insert at end of *TPC*(*T*) the first variable in *OPEN* and remove it from *OPEN*   Elimination strategy  If *OPEN* = ∅  For each *X* ∈ *TPC*(*T*)  If  ***Z*** ⊆ *TPC*(*T*) \ {*X*}, such that *X* is conditionally independent of *T* given ***Z***, then remove *X* from *TPC*(*T*)  Else  *X* 🡨 last variable added to *TPC*(*T*)  If  ***Z*** ⊆ *TPC*(*T*) \ {*X*}, such that *X* is conditionally independent of *T* given ***Z***, then remove *X* from *TPC(T)*  Interleaving strategy  Repeat  steps #2 and #3 of GLL-PC-nonsym  Until *OPEN* = ∅ |
| --- |
